# Ultimate brightness of a medium-energy synchrotron light source at operational beam intensity

**DOI:** 10.1107/S1600577525002723

**Published:** 2025-04-23

**Authors:** Victor Smaluk, Timur Shaftan, Dean Hidas

**Affiliations:** a Brookhaven National Laboratory Upton NY11973 USA; SESAME, Jordan

**Keywords:** synchrotrons, brightness, undulator radiation, emittance, intensity-dependent effects

## Abstract

Synchrotron light sources are key instruments of modern science, providing unique opportunities for groundbreaking studies in diverse scientific disciplines and driving innovation in numerous scientific and technological fields. Fourth-generation light sources provide unprecedented capabilities in imaging, spectroscopy and diffraction techniques. Ultimate brightness is the key to advancing to a smaller scale, faster response, and higher data measurement and processing rate. The brightness is primarily determined by the electron beam emittance and energy spread at operational intensity. A common feature of fourth-generation synchrotrons is the short length of the electron bunches combined with a very small transverse beam size. Consequently, the high particle density leads to strong collective effects that significantly increase the emittance and limit the achievable brightness at operational beam intensity. In this article, we summarize our studies of the emittance and brightness scaled with the beam energy and intensity, taking into account the effects of intrabeam scattering, beam-impedance interaction and bunch lengthening provided by higher-harmonic RF systems to identify optimal combinations of machine and beam parameters.

## Abstract 2

Recent studies of the ultimate brightness limits of medium-energy synchrotron light sources at operational beam intensities are summarized, focusing on how collective effects, such as intrabeam scattering and bunch lengthening, influence beam emittance and brightness. The study identifies optimal beam and machine parameters that balance these effects to maximize brightness for user experiments.

## 1. Introduction

**Figure 1:**
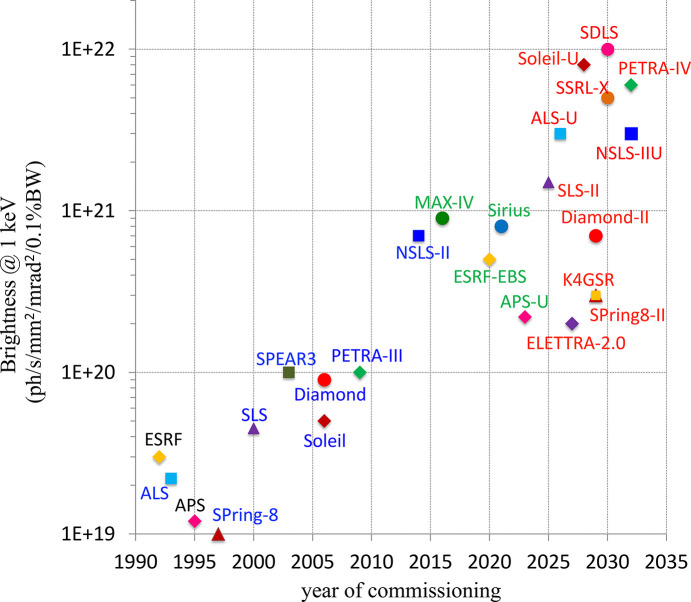
Evolution of brightness at 1 keV photon energy.

Spectral brightness, defined as the number of photons per second per unit phase space area within a 0.1% bandwidth of photon energy, is a practical figure of merit for light sources. High-brightness X-ray beams are demanded by numerous areas of scientific research, *e.g.* imaging the dynamics of materials on their relevant functional time and length scales, including studies on rechargeable Li-ion batteries, room-temperature crystallography of large molecules within sub-millisecond time scales and other advanced applications. A consistent increase in brightness has marked the evolution of synchrotrons over the past few decades. For the two most popular values of photon energy, 1 keV (soft X-ray users) and 10 keV (hard X-ray users), Figs. 1 ▸ and 2 ▸ show the maximum brightness among major light source projects in the past 30 years, as well as the estimates for future projects in the next decade. As one can see, all projects of new and upgraded synchrotrons aim to achieve a substantial increase in brightness, spanning several orders of magnitude compared with third-generation light sources. The brightness-relevant parameters of the light sources presented in the figures are summarized in Table 1 ▸: energy *E*, circumference *C*, average beam current *I*, horizontal ɛ_*x*_ and vertical ɛ_*y*_ emittance, relative beam energy spread σ_δ_, and horizontal 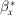 and vertical 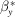 beta functions in the centers of straight sections where undulators are installed. All the data were taken from the published materials listed in Table 1 ▸. Note that the published graphs of brightness are not always accompanied by detailed lists of undulator and beam parameters, so we had to look for those parameters in other publications. For a consistent comparison, we use the values of emittance and energy spread determined by magnet lattices without insertion devices. However, the effects of damping wigglers are included for NSLS-II, MAX-IV, PETRA-IV, SPring8-II, SSRL-X and SDLS because those wigglers are a part of the baseline lattice design. Collective effects of beam dynamics, such as intrabeam scattering and impedance-driven bunch lengthening, are not taken into account, nor is the bunch lengthening provided by higher-harmonic cavities. Analysis of these effects can be found in some of the cited publications. If the horizontal and vertical emittances were not explicitly specified, we calculated them from the natural emittance and coupling. The beta functions in the centers of straight sections were estimated from the graphs if not available in publications. For the NSLS-II upgrade, we scaled the emittance with energy from 3 to 4 GeV and included the effect of three pairs of damping wigglers installed as a part of the NSLS-II project.

In modern synchrotrons, undulators of various types are the brightest and most commonly used light-generating devices. The theory of undulator radiation is well developed and has been published in journal articles and textbooks, *e.g.* Kim (1989 ▸), Hofmann (2004 ▸) and Clarke (2004 ▸). The on-axis brightness of undulator radiation is proportional to the photon flux in the photon energy bandwidth and the phase space volume determined by the transverse sizes and divergences of the electron and photon beams. The root-mean-square (r.m.s.) size and divergence of the electron beam are determined by its emittance and the beta functions in the undulator, with a contribution from the energy spread and dispersion. To calculate the r.m.s. size and divergence of the photon beam considering the energy spread, analytical formulae were derived by Tanaka & Kitamura (2009 ▸), but their applicability near the diffraction limit has been questioned (Walker, 2019 ▸).

Advanced simulation codes (Chubar *et al.*, 2011 ▸; Hidas, 2018 ▸) have been developed for a comprehensive analysis of undulator radiation, including calculations of angular distribution, coherent modes, power density and other relevant parameters. For accurate calculations of the brightness discussed in Section 5, we used the well established code *Synchrotron Radiation Workshop* (*SRW*) (Chubar *et al.*, 2011 ▸; Nash *et al.*, 2019 ▸; Rakitin *et al.*, 2018 ▸).

The photon flux is proportional to the electron beam intensity, so, in principle, one can get a higher brightness by increasing the average beam current. However, technical problems, such as high RF power, beam-induced heating and collective instabilities, significantly limit this approach. No light sources are operating with the beam current exceeding 500 mA. Reducing the electron beam emittance and matching beta functions in undulators by advancing magnet lattice design is an efficient way to increase brightness.

## 2. Evolution of beam emittance

The natural emittance of an electron beam in a storage ring is determined by a balance of radiation damping and quantum excitation. For a planar ring without vertical bending, this relation is expressed as a ratio of radiation integrals, 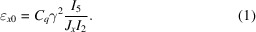
Here, ɛ_*x*0_ is the emittance, γ is the Lorentz factor, *C*_*q*_ = 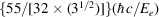 ≃ 3.83 × 10^−13^ m, *J*_*x*_ is the horizontal damping partition number, and *I*_2_ and *I*_5_ are the radiation integrals (Helm *et al.*, 1973 ▸). The emittance can be represented in a simple way as 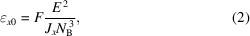
where *F* is some function of the magnet lattice, *E* is the electron energy and *N*_B_ is the number of bending (dipole) magnets in the ring.

**Figure 3:**
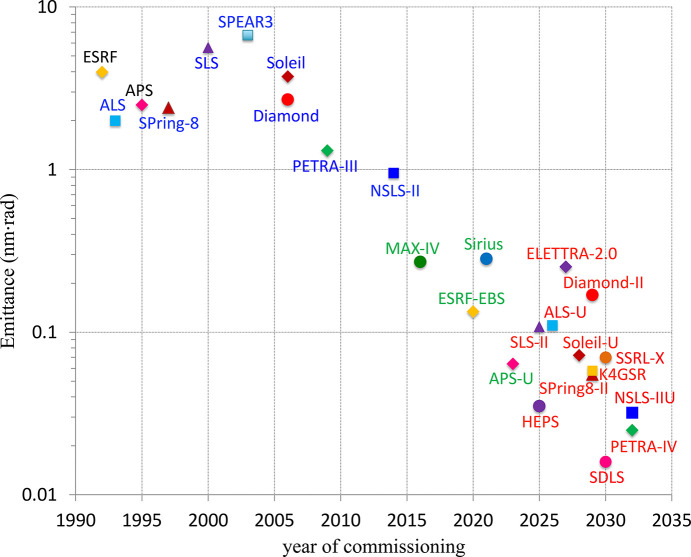
Evolution of the electron beam emittance in synchrotron light sources.

Since the emittance is inversely proportional to the cube of the number of bending magnets, increasing *N*_B_ is the most efficient way of designing a low-emittance lattice. As a result, we see a transition from the double-bend and triple-bend achromat lattices used to build third-generation light sources worldwide to the multi-bend achromat (MBA), which is the basic lattice option for new light sources. Four MBA-based synchrotrons have been commissioned in the past eight years: MAX-IV (Sweden, 2016), ESRF-EBS (France, 2020), SIRIUS (Brazil, 2020) and APS-U (USA, 2024). Many other projects of new and upgraded facilities are being developed worldwide, *e.g.* ALS-U (USA), HEPS (China), Elettra-2 (Italy), Diamond-II (UK), Soleil-2 (France), PETRA IV (Germany), CLS-2 (Canada), Korea-4GSR and others. The beam emittance has been continuously reduced over a few decades of synchrotron development, as illustrated by Fig. 3 ▸.

There is a recent trend in magnet design towards combined magnets with field profiles tailored to the lattice requirements. In the near future, we expect a transition to permanent-magnet bending/focusing elements, providing high quadrupole gradients, saving space and reducing total power consumption. The use of superconducting high-field high-gradient magnets also looks promising for future projects.

A new approach of a low-emittance lattice design alternative to MBA has recently been proposed at NSLS-II, Brookhaven National Laboratory: use of a new lattice element ‘complex bend’ replacing regular dipole magnets (Shaftan *et al.*, 2018*a* ▸; Wang *et al.*, 2018 ▸; Shaftan *et al.*, 2018*b* ▸; Wang *et al.*, 2019 ▸). The main advantage of the complex bend design is to enable many more dense dipoles integrated into the same element. Since the emittance scales inversely as the cube of the number of dipoles, this opens the possibility of achieving gains in emittance. For example, the replacement of the dipole magnets with complex bends in the NSLS-II DBA lattice, keeping the layout of matching quadrupole triplets and straight sections unchanged, results in an emittance reduction of a factor of 30 (Smaluk & Shaftan, 2019 ▸), from 2050 pm to 65 pm (‘bare’ lattice without wigglers and undulators). However, the required quadrupole gradient of the complex bend magnets was about 200 T m^−1^, exceeding the practical limit of using permanent magnets (about 130 T m^−1^). We decided to explore options of whole ring replacement, limiting the quadrupole gradient below 130 T m^−1^ to use complex bends based on permanent magnets, which significantly reduces the manufacturing and operation costs. Advanced options of the complex bend lattice design for the NSLS-II upgrade provide an emittance of about 24 pm (Plassard *et al.*, 2021 ▸; Song & Shaftan, 2024 ▸) with optimized beta functions in the undulators and the lengths of the straight sections almost the same as in the present NSLS-II.

## 3. Energy and intensity constraints

The major practical limit of the electron beam current *I*_b_ and energy *E* in a storage ring results from the synchrotron radiation power increasing rapidly with the beam energy as *E*^4^, 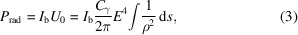
where *C*_γ_ = 8.846 × 10^−5^ m GeV^−3^, *U*_0_ is the radiation energy loss per turn and ρ is the bending radius.

In modern synchrotrons, the total radiation power is dominated by the light-generating insertion devices (wigglers and undulators), the contribution of which is usually higher than the contribution of the dipole magnets. The beam energy loss caused by radiation is compensated by complex and expensive RF systems, which may contribute a significant part of the total cost of the machine construction and operation due to their high power consumption, depending on specific projects and sites.

**Figure 4:**
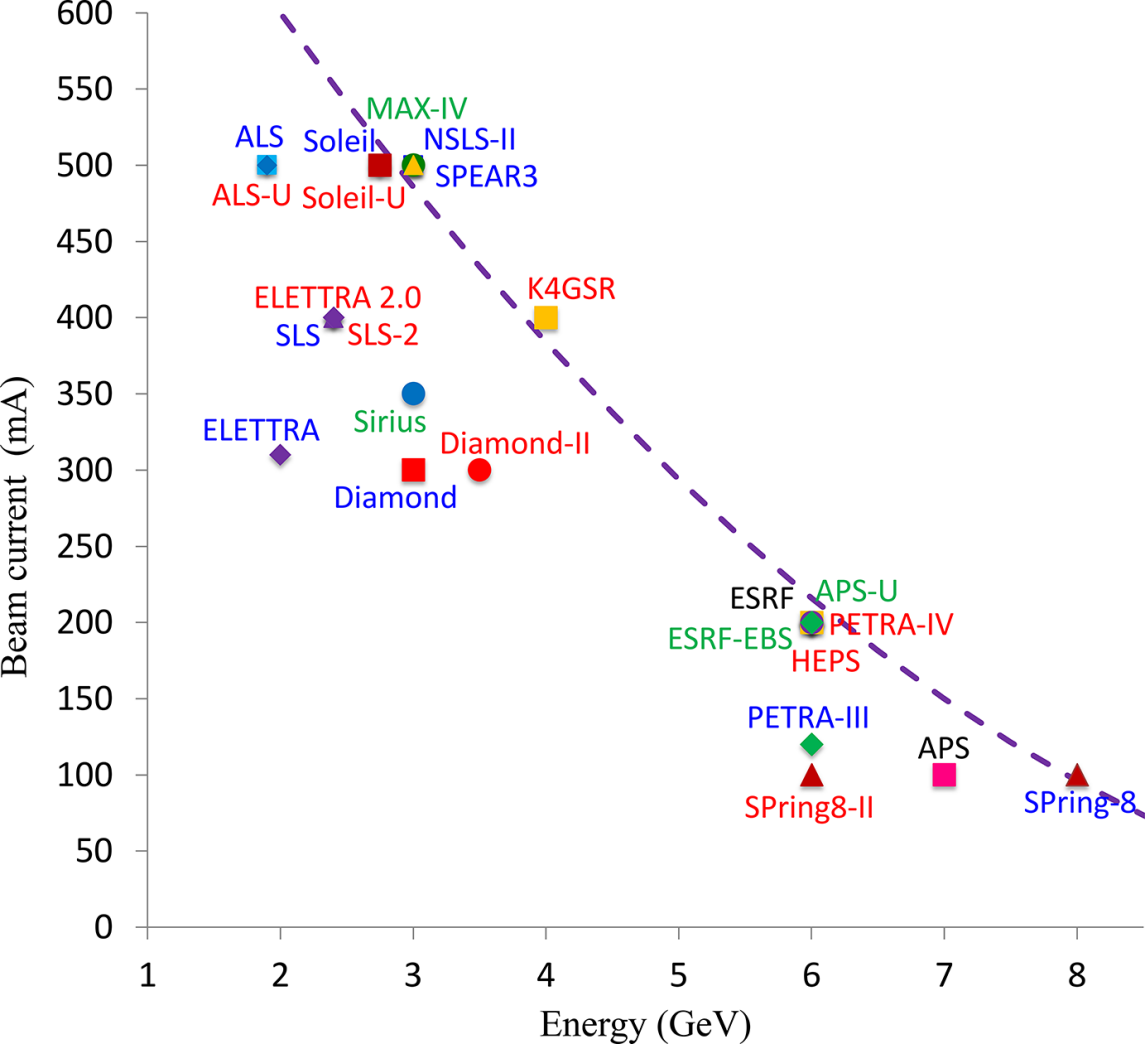
Design electron beam current versus energy.

Mainly for the above-mentioned reason, higher-energy synchrotrons operate with lower beam intensity, as illustrated in Fig. 4 ▸, which shows the design electron beam current of several facilities in operation and projects under development. All the data are taken from publications listed in Table 1 ▸. The dashed curve represents an empirical energy-dependent limit for the beam current. Note that synchrotron light sources often need some time after commissioning to start routine user operations with the design beam intensity. For example, NSLS-II demonstrated the design beam current in 2019 and started part-time user operations with 500 mA even later.

Another important factor that could limit the beam intensity is beam-induced heating of the vacuum chambers, which is directly proportional to the longitudinal impedance (mainly the resistive-wall one) and the square of the beam current. Strong focusing magnets and high-brightness insertion devices require low-aperture vacuum chambers. Since the longitudinal impedance is inversely proportional to the vacuum chamber size, small vacuum chambers and short bunch lengths lead to higher beam-induced power. The transverse impedance is inversely proportional to the cube of the vacuum chamber size. The beam interaction with the impedance can also lead to beam instabilities, further limiting the maximum stable beam current. Interaction with residual gas in the vacuum chamber can excite instabilities too, and this problem is also more severe for fourth-generation synchrotrons because the vacuum chambers are smaller and pumping is more difficult.

There are several ways of mitigating the challenges caused by these collective effects. Lattice optimization can help to increase the natural bunch length. Bunch lengthening is essential to reduce the peak current of the beam and can be achieved by implementing advanced schemes for higher-harmonic RF cavities (HHC). For a fixed ring circumference and total beam current, increasing the number of bunches is also helpful in reducing the peak current. However, this reduction is rather limited when the bunch lengthening by harmonic cavities is taken into account. To reduce the impedance, a larger vacuum chamber should be used where possible, and smooth transitions must be implemented. Minimization of the impedance needs to be a part of the vacuum chamber design from the very beginning of a project.

## 4. Intensity-dependent emittance

Without the collective effects of electron beam dynamics, the photon flux and brightness should be directly proportional to the average beam current. However, these collective effects at operational beam intensity play a crucial role in determining the practically achievable performance of light sources. In modern low-emittance rings, electron beams are small in all three dimensions: a small momentum compaction results in a short bunch length, while a low emittance determines small transverse sizes.

**Figure 5:**
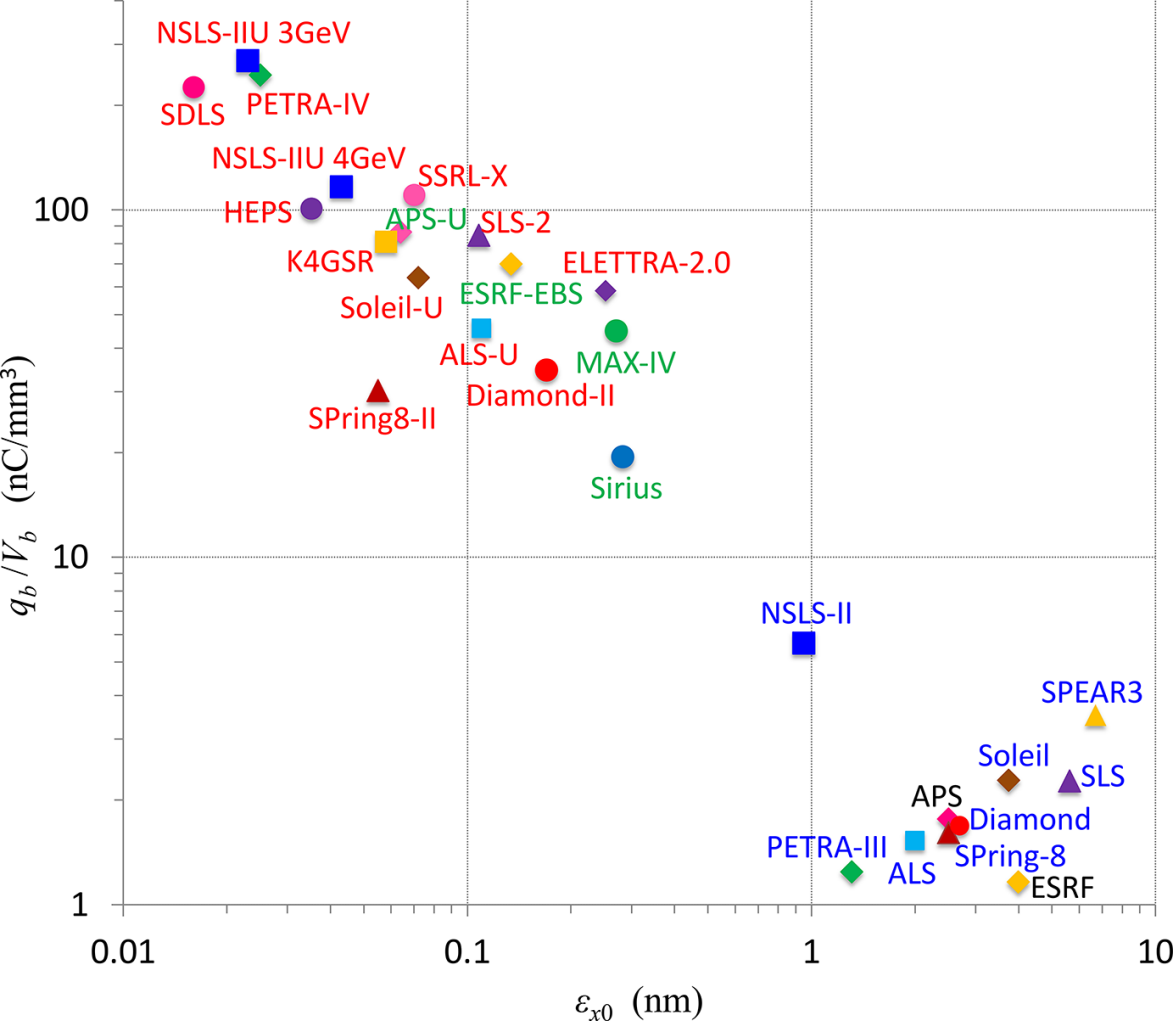
Charge density versus electron beam emittance.

Fig. 5 ▸ demonstrates the charge density *q*_b_/*V*_b_ as a function of the emittance for a set of synchrotrons worldwide, in operation or under development. A trend of a significant increase in the particle density within the bunch in low-emittance rings is clearly seen, resulting in much stronger collective effects. Here *V*_b_ = (4π^3^)^1/2^σ_*x*_σ_*y*_σ_*z*_ is the bunch volume, σ_*z*_ is the r.m.s. bunch length and σ_*x*,*y*_ is the horizontal/vertical beam size. Note that the bunch volume was calculated at zero beam intensity and without the higher-harmonic cavities widely used to increase the bunch length for mitigation of collective effects, so the bunch length σ_*z*_ is equal to σ_*z*0_ completely determined by the lattice and RF parameters: 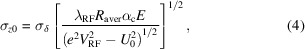
where σ_δ_ is the relative energy spread, λ_RF_ is the RF wavelength, *V*_RF_ is the RF voltage, *R*_aver_ is the average ring radius and α_c_ is the momentum compaction factor.

Intrabeam scattering (IBS) is one of the adverse effects that can impact beam quality and impose limitations on the ultimate performance of low- and medium-energy synchrotrons (Borland, 2012 ▸; Steier *et al.*, 2016 ▸; Huang, 2017 ▸; Blednykh *et al.*, 2021 ▸). Since IBS is a small-angle scattering, it does not cause particle loss but results in a substantial intensity-dependent increase in emittance, energy spread and bunch length. The theory of IBS has been well developed for quite some time (Piwinski, 1974 ▸; Bjorken & Mtingwa, 1983 ▸; Bane, 2002 ▸; Kubo *et al.*, 2005 ▸) and has been implemented into particle tracking codes (Borland, 2000 ▸).

We employed the high-energy approximation of the IBS theory (Bane *et al.*, 2001 ▸) to examine the combined effect of IBS and the bunch lengthening resulting from the longitudinal impedance and higher-harmonic cavities. The equilibrium emittances ɛ_*x*,*y*_ and relative energy spread σ_δ_ at the beam current *I*_b_ are expressed as follows: 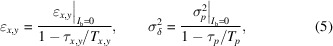
where τ_*x*_, τ_*y*_ and τ_*p*_ are the radiation damping times, and *T*_*x*_, *T*_*y*_ and *T*_*p*_ are the IBS growth times, 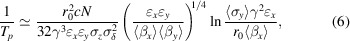

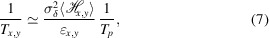
*N* = 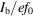 is the number of electrons per bunch, *f*_0_ is the revolution frequency, *r*_0_ is classical electron radius and 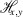 = 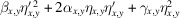 is a function determined by the lattice.

In a practical range of beam energy and current, we analyzed the impact of intensity-dependent effects on light source performance using the complex bend lattice for the NSLS-II upgrade (Song & Shaftan, 2024 ▸) optimized to achieve a minimum emittance, and a decent dynamic aperture and beam lifetime. The light-generating insertion devices (IDs) contribute significantly to the total energy loss per turn *U*_0_, determining the radiation damping, emittance and energy spread. Thus, for the NSLS-II upgrade lattice with a full set of IDs, the total energy loss is about 1 MeV, compared with 0.2 MeV caused by bending magnets only. For this lattice, we calculated the emittance as a function of the beam current and energy, considering collective effects. We assumed a 100% betatron coupling κ = ɛ_*y*_/ɛ_*x*_ = 1. Since the beta functions at the centers of straight sections are almost equal, this corresponds to the round-beam operation mode.

**Figure 6:**
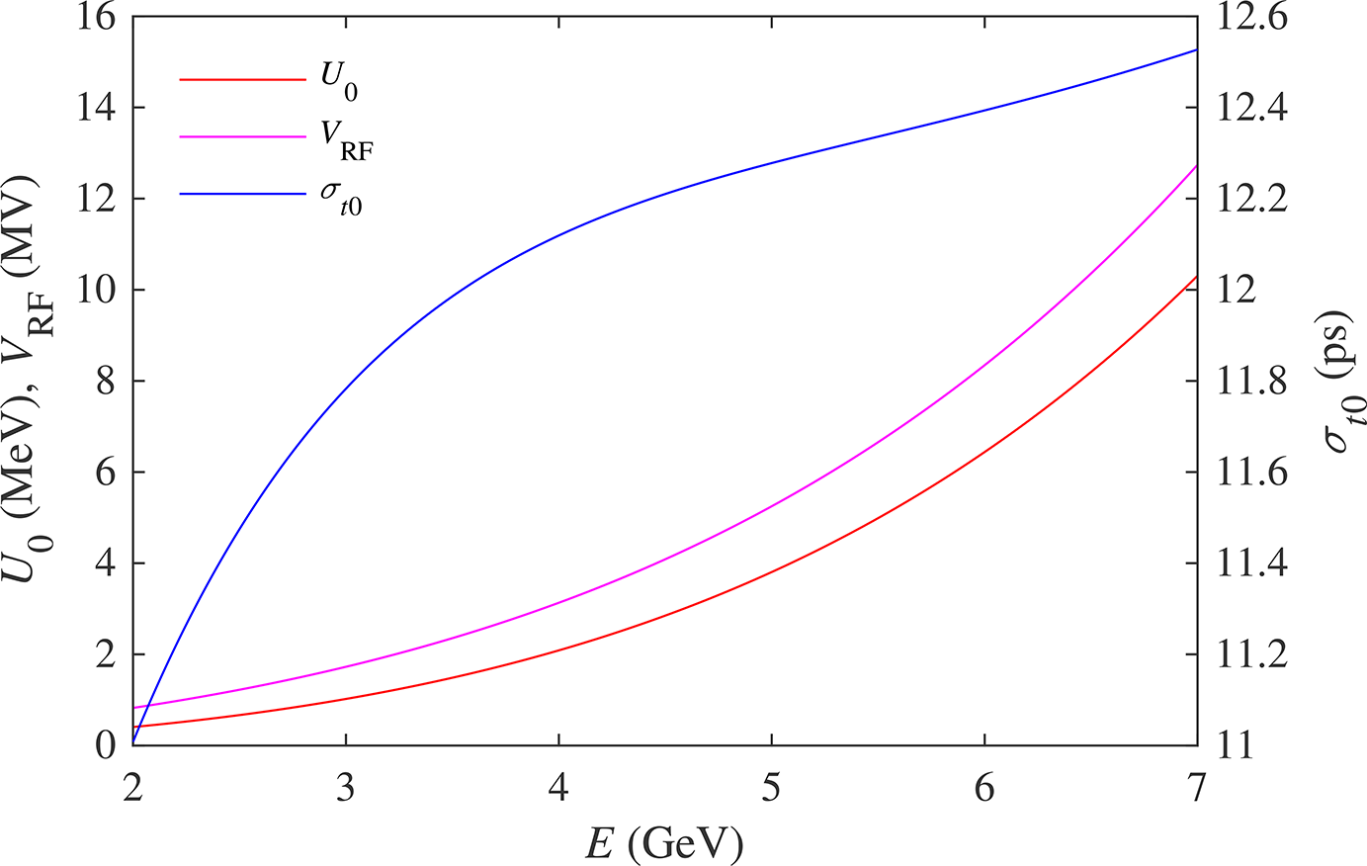
Radiation energy loss, RF voltage and the zero-intensity bunch length.

The RF voltage was scaled with the energy to keep the RF acceptance of ±3%, which is required to match the momentum aperture of the lattice. Fig. 6 ▸ shows the radiation energy loss per turn *U*_0_, RF voltage *V*_RF_ and zero-intensity bunch length σ_*z*0_ [equation (4)] as functions of energy.

The bunch lengthening caused by the beam interaction with the longitudinal impedance was computed at each iteration using the modified Zotter equation (Zotter, 1981 ▸; Zhou *et al.*, 2023 ▸),
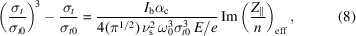
where ν_s_ = ω_s_/ω_0_ is the synchrotron tune, σ_*t*_ = σ_*z*_/*c* and σ_*t*0_ is the bunch length at zero intensity. A typical effective normalized longitudinal impedance Im(*Z*_∥_/*n*)_eff_ = 0.5 Ω was assumed. We limited the beam-impedance interaction by the simplified model (8) in the absence of a detailed frequency-dependent impedance at the present stage of the machine design. We assumed the operational bunch current to be below the threshold of microwave instability, causing a growth in the energy spread. However, for a detailed analysis of any specific lattice and impedance, multi-particle tracking simulations, including total wake fields, will be required.

We used modified equations (5)–(7) to calculate the IBS-driven emittance growth with full coupling (ɛ_*y*_ = ɛ_*x*_), together with equation (8) in a differential form to take the impedance-driven bunch lengthening into account. Equations (5)–(8) were solved iteratively, increasing the beam intensity in small steps. The analytical calculations were benchmarked with the multi-particle tracking carried out using the *ELEGANT* code (Borland, 2000 ▸).

We considered the use of higher-harmonic RF cavities providing bunch lengthening to mitigate collective effects; the effect of HHCs was simply modeled by multiplying the zero-intensity bunch length σ_*z*0_ by a bunch lengthening factor. More realistic modeling of HHC effects on beam dynamics needs comprehensive numerical simulations taking into account the beam loading and stability, but this is outside the scope of this article.

**Figure 7:**
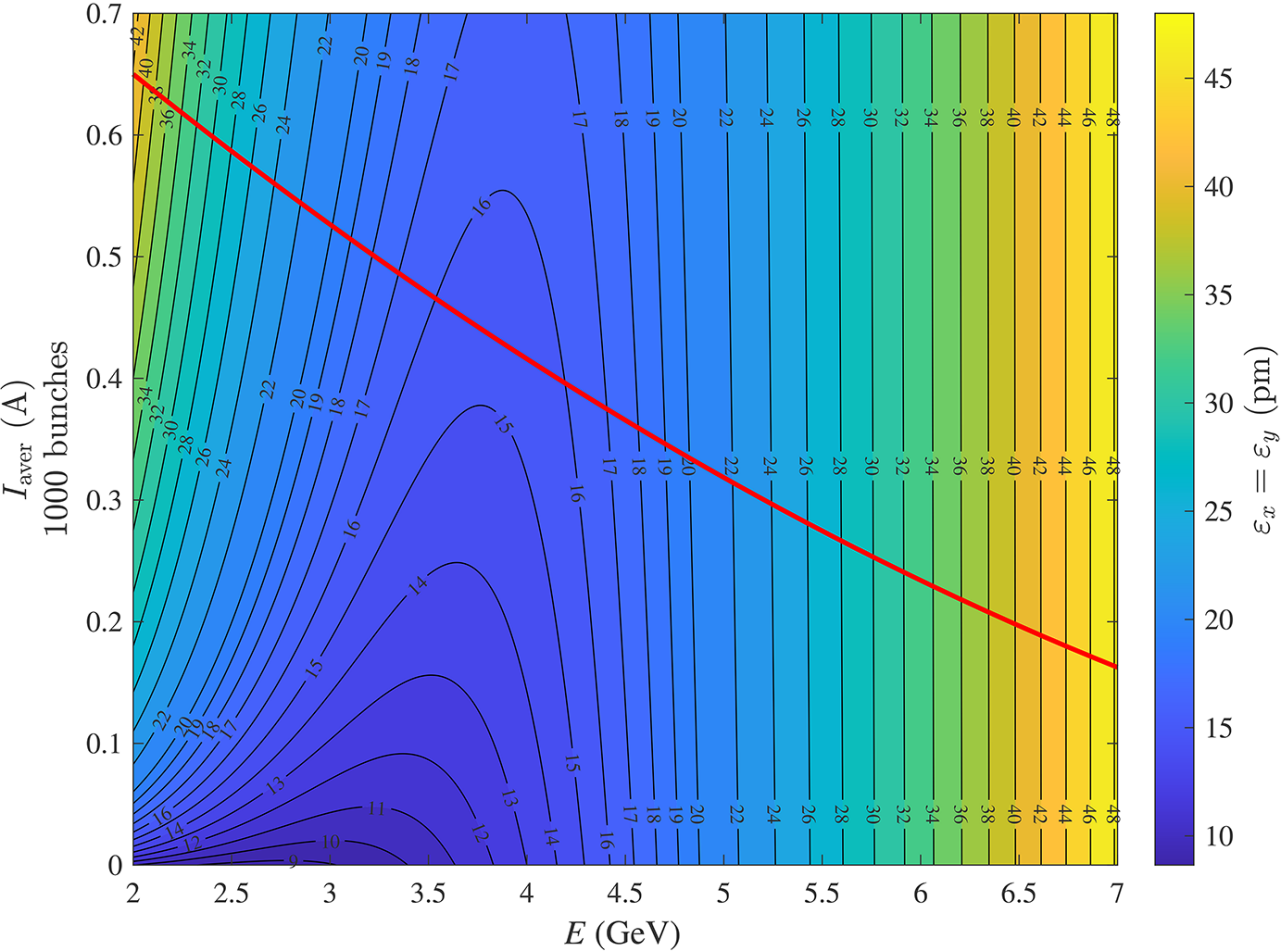
Combined effect of IBS, impedance and higher-harmonic cavities on the beam emittance. The red curve represents the empirical beam intensity limit from Fig. 4 ▸.

With all the above-mentioned collective effects and a moderate HHC bunch lengthening factor of 3, we calculated the emittance as a function of the energy *E* and average beam current *I*_aver_ uniformly distributed in 1000 bunches; the results are presented in Fig. 7 ▸. We found that the emittance at the operational beam intensity reaches a minimum in the energy range of 3.5–4 GeV. This is due to the strong emittance blow-up increase caused by IBS at lower energies, while the *E*^2^ factor in equation (2) leads to an increase in emittance at higher energies.

**Figure 8:**
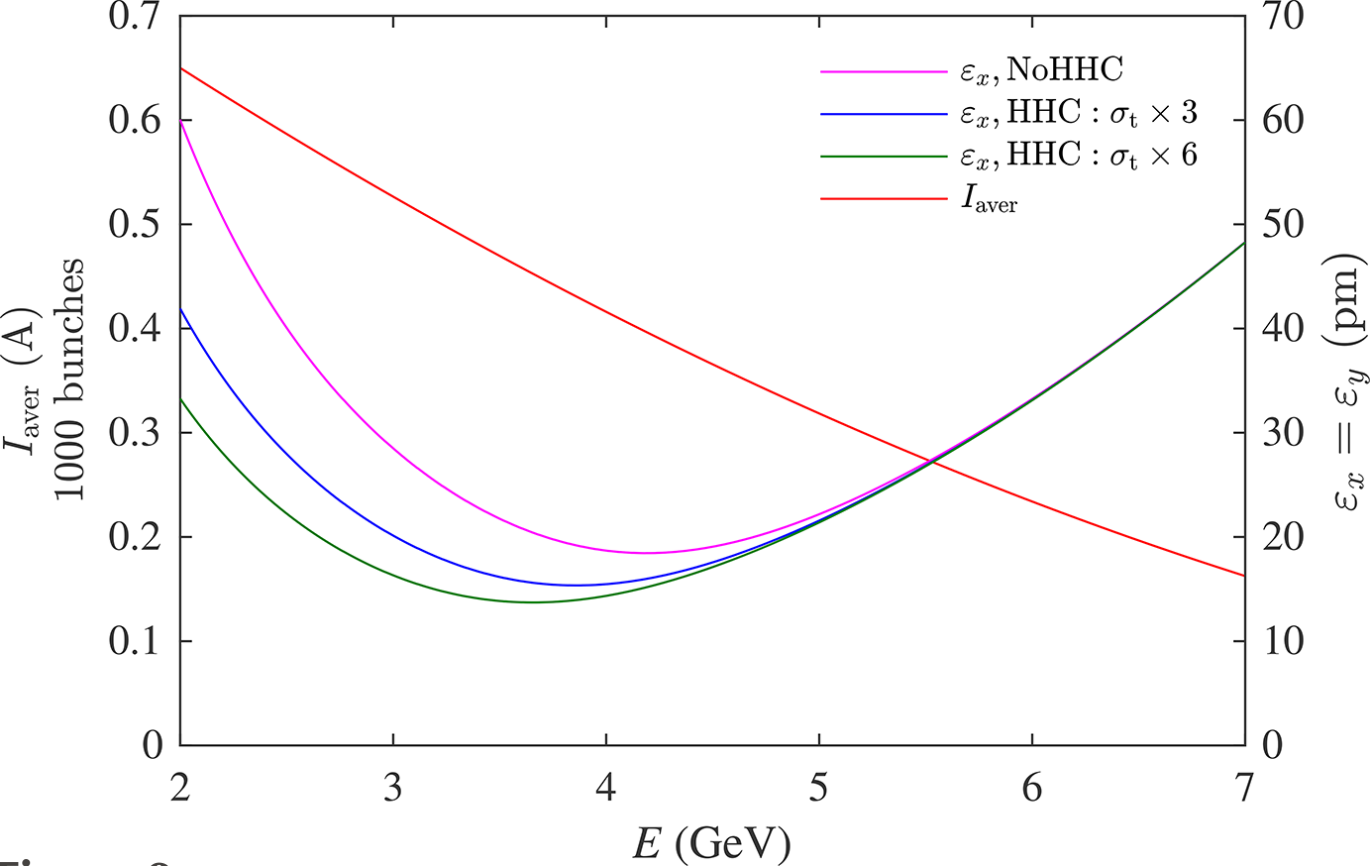
Bunch lengthening effect on the IBS-driven emittance growth.

Note that larger HHC bunch lengthening factors will further mitigate IBS and reduce the emittance at lower energy. As an example, Fig. 8 ▸ shows the emittance at the energy-dependent beam current for the NSLS-IIU complex bend lattice (Song & Shaftan, 2024 ▸), assuming bunch lengthening factors of 1 (no HHC), 3 and 6. As one can see, the more efficient bunch lengthening provided by harmonic RF systems pushes down the energy that maximizes brightness. This is why we consider a combination of higher-harmonic cavities with different harmonic numbers for the NSLS-II upgrade to reach a bunch lengthening factor of 5 or higher.

**Figure 9:**
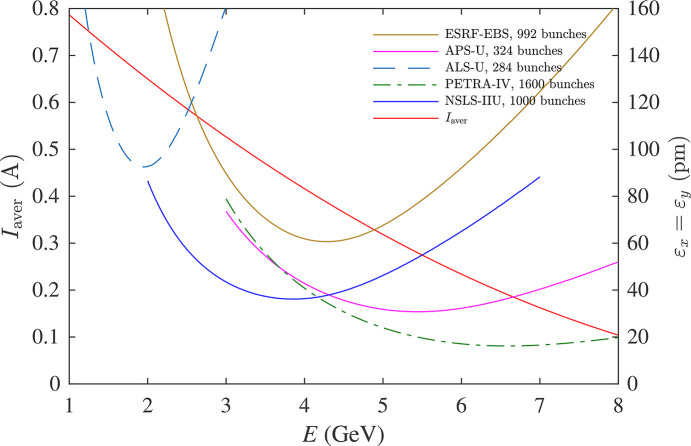
IBS-affected emittance of low-emittance synchrotrons (‘bare’ lattices, 100% coupling).

Thus, the optimal energy point with the smallest operational emittance is determined by a specific design of the lattice and RF system, including higher-harmonic cavities, and it is also affected by wigglers and undulators. For illustration, we calculated the IBS-affected emittance as a function of energy for five low-emittance synchrotrons: ESRF-EBS, APS-U, ALS-U, PETRA-IV and NSLS-IIU. The emittance was calculated using the *ibsEmittance* function, which is a part of the *ELEGANT* package, assuming ‘bare’ lattices without wigglers and undulators and 100% coupling (ɛ_*y*_ = ɛ_*x*_). The results are summarized in Fig. 9 ▸, where the curves represent the emittance at the energy-dependent beam current *I*_aver_, with the design number of bunches specified for each facility.

The impact of higher ring energy on the operating cost is profound and scales sharply with the former. The bulk of the additional cost for higher energy resides in elevated requirements for RF systems that scale as energy to the fourth order, constraining the available beam intensity. Therefore, a ring energy of 3.5 GeV calls for 85% more power than one of 3 GeV. An RF system for a 4 GeV ring would consume 310% more power than for 3 GeV at the same beam current, significantly increasing the energy bill for the facility. Separately, the magnet parameters (fields and gradients) would need to be higher (scaling proportionally to the beam energy), which elevates the cost of magnets, coils, power supplies, cabling and cooling, significantly contributing to the power bill of the facility as well.

## 5. Ultimate brightness

Using the *SRW* code, we calculated the brightness at photon energies of 1 keV and 10 keV as a function of the beam current and energy for optimized in-vacuum undulators (IVU), taking into account all collective effects discussed above: intrabeam scattering, and bunch lengthening caused by higher harmonic RF cavities and longitudinal impedance. This optimization was done at each point in the 2D maps shown in Fig. 7 ▸.

The IVU peak fields *B*_*y*_ as a function of the ratio of the magnetic gap over the period 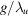 are estimated from equation (9) using the coefficients from Elleaume *et al.* (2000 ▸) for a hybrid magnetic structure with vanadium permendur (*a* = 3.694, *b* = −5.068, *c* = 1.520),
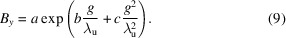
This field estimate is in good agreement with currently installed IVUs at NSLS-II. We consider the period λ_u_ to range from 15 mm to 50 mm with a granularity of 0.25 mm and undulator harmonics *n* up to 21. For every period and harmonic in this range, we calculate the required magnetic field *B*_0_ using the equations 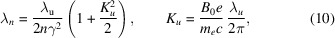
for 1 keV and 10 keV emission (if possible), then solve equation (9) for the required magnetic gap. The IVU magnetic length *L*_u_ is taken to be the maximum allowable, assuming a stay-clear of 1000 times the vertical beam size at the extremities and assuming a maximum cutoff at 6.8 m. This definition is just a proxy for the vertical acceptance, which simply follows the vertical beam profile. We think it is a conservative initial assumption and would accommodate even off-axis injection schemes with full coupling. This will be studied in detail once the lattice design is finalized.

**Figure 10:**
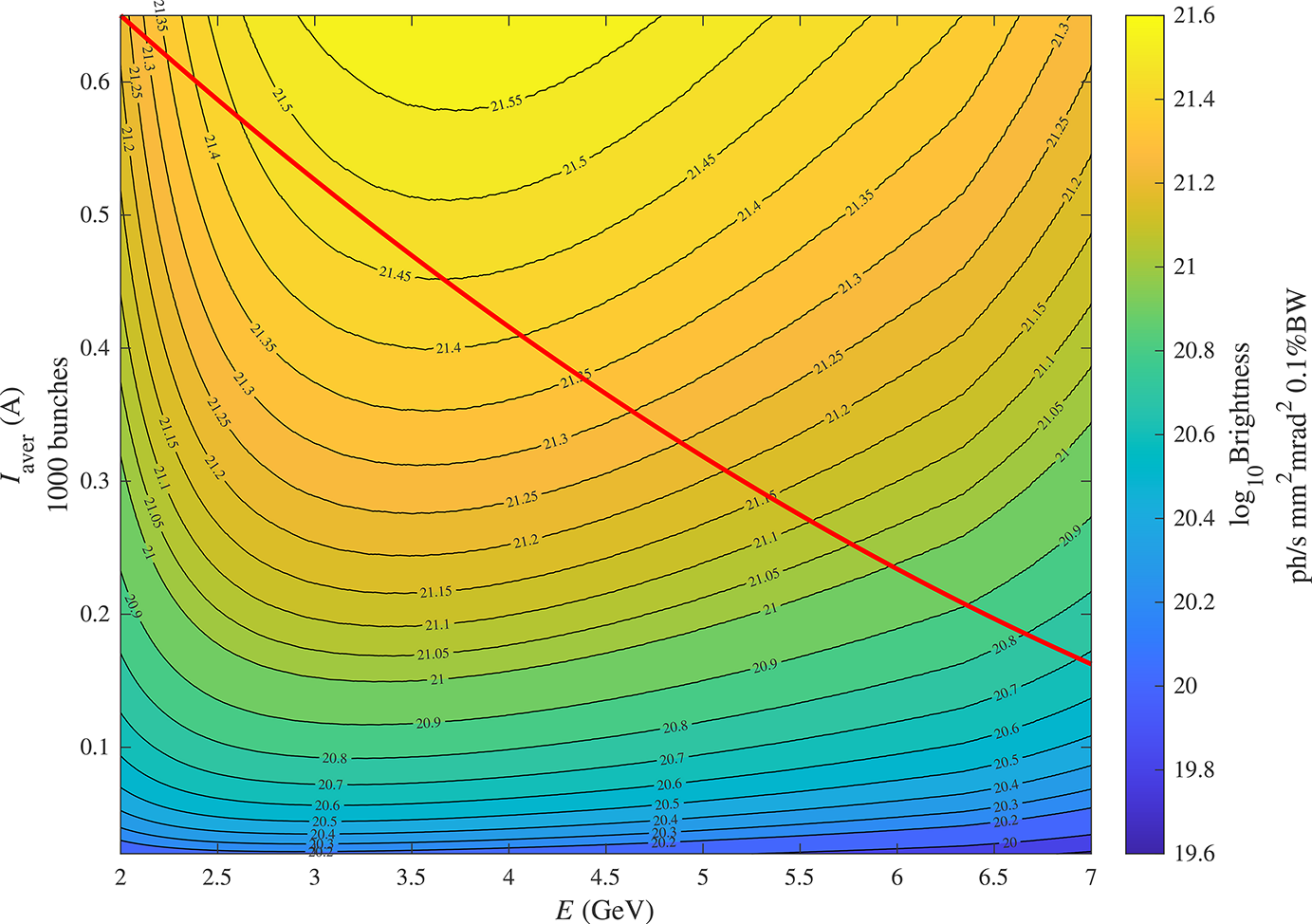
Brightness of an optimized IVU at 1 keV photon energy. The red curve represents the empirical beam intensity limit from Fig. 4.

**Figure 11:**
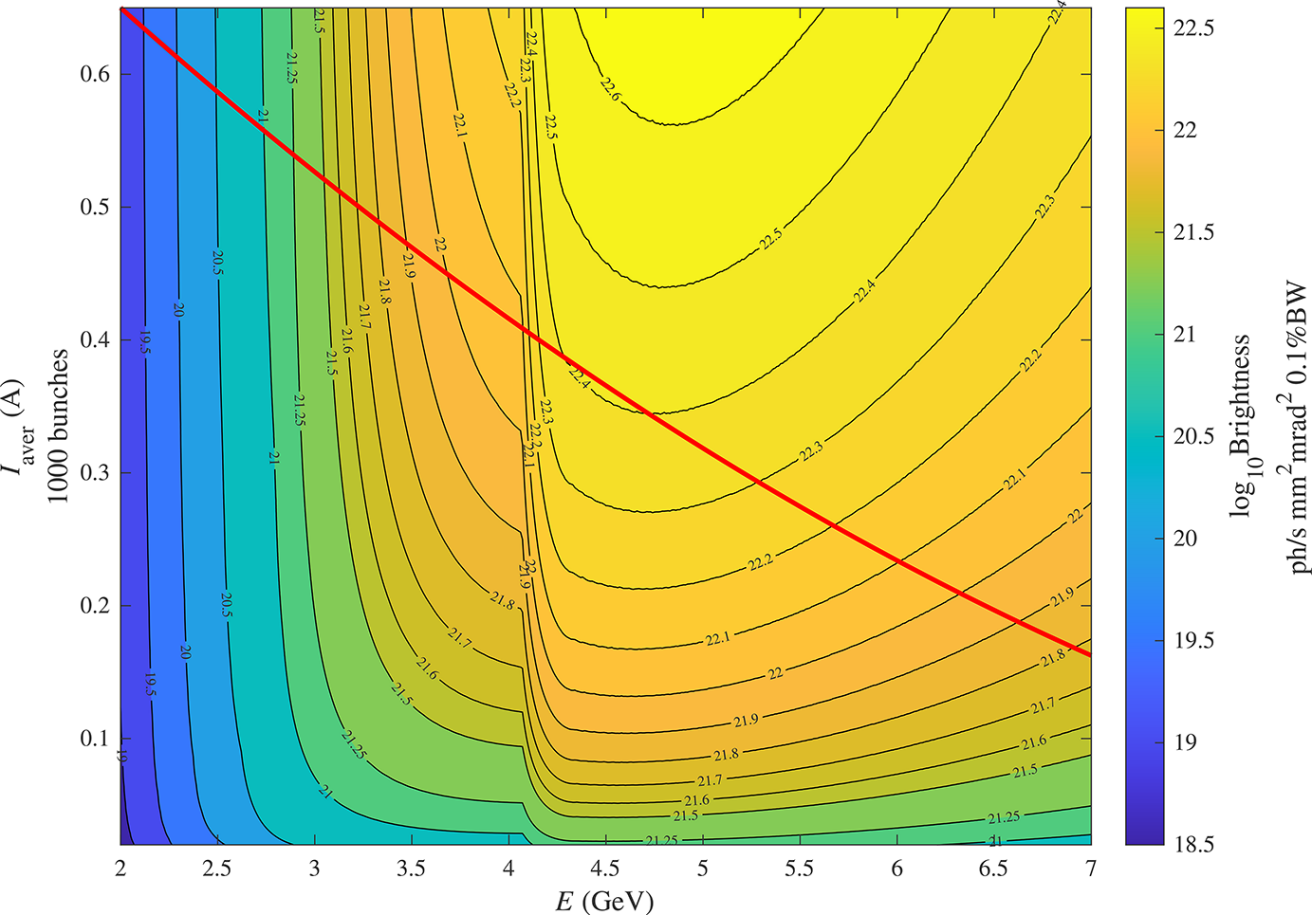
Brightness of an optimized IVU at 10 keV photon energy. The red curve represents the empirical beam intensity limit from Fig. 4.

For photon energies of 1 keV and 10 keV, the maximum brightnesses calculated according to this procedure are shown in Figs. 10 ▸ and 11 ▸, respectively, as a function of the beam energy and current. There is a sharp transition in Fig. 11 ▸, which occurs at 4.06 GeV and is due to a transition from the third to the first harmonic. For the undulator with a period of 15 mm, the tail of the first-harmonic brightness curve begins to reach 10 keV at 4 GeV electron energy but does not become dominant over the third harmonic of longer-period devices (22 mm in this case) until 4.06 GeV, above which the first harmonic will then always have a higher brightness. The sharpness of this transition is a direct result of the minimum period considered. There are other harmonic transitions less noticeable at lower beam energies in Fig. 11 ▸. For the photon energy of 1 keV, there are no such transitions in Fig. 10 ▸ because the first-harmonic brightness is maximal in the whole range.

## 6. Conclusions

The brightness of X-ray beams, which is a practical figure of merit for light sources, has shown fast growth in past decades. Fourth-generation synchrotrons have a common feature of short electron bunches and a small transverse beam size, resulting in a significant reduction in bunch volume, higher particle density and stronger collective effects of electron beam dynamics. The maximum brightness at operational beam intensity is significantly affected by collective effects determining the practically achievable performance of light sources, especially at lower electron energies. With a given beam intensity limited by technical constraints, the brightness is predominantly determined by the electron beam emittance. To identify optimal combinations of machine and beam parameters, we studied the emittance scaling with the beam intensity and energy, considering the effects of intrabeam scattering, beam-impedance interaction and bunch lengthening by higher-harmonic RF cavities.

We limit the beam-impedance interaction by a simplified model of potential well distortion, although for more detailed analysis multi-particle tracking simulations will be required. The effect of higher-harmonic cavities was simply modeled by multiplying the zero-intensity bunch length by a lengthening factor. This approach is adequate for our purposes, but more realistic modeling needs comprehensive numerical simulations taking into account the beam loading and stability.

For the complex bend lattice designed for the high-brightness NSLS-II upgrade, we found a specific energy where the intensity-dependent emittance reaches a minimum due to the interplay between the quadratic increase in the zero-intensity emittance with energy and the IBS-induced increase at lower energies. Specific lattice and RF parameters determine this optimal energy point with the smallest operational emittance, and larger bunch lengthening provided by higher-harmonic cavities moves this optimal point to a lower energy.

For the most popular photon energies, 1 keV (soft X-ray users) and 10 keV (hard X-ray users), we calculated the brightness as a function of the beam current and energy for optimized in-vacuum undulators, taking into account all the collective effects discussed above. The ultimate brightness is determined by the minimum operational emittance and parameters of the undulators optimized for a specific photon energy. Longer undulators with smaller gaps generate brighter beams, but the maximum available length of straight sections to place undulators and the minimum possible gaps are limited by the lattice design. So for higher brightness, not only minimum emittance but also long enough undulator sections with proper beta functions are essential.

There is a notable correlation between brightness at a particular photon energy and many of the parameters discussed in this paper, notably electron beam energy and straight section lengths. The tender to hard X-ray range sees overall gains in brightness with higher beam energies; there is no doubt that maintaining brightness and flux at very low photon energies is more difficult as the electron beam energy increases, and longer straight sections for this would be desirable. At a photon energy of 10 keV, however, this is generally not a limiting factor, as competing constraints will dominate. At 10 keV, the length is only a limiting factor at about 6.4 GeV and around the first to third harmonic transition. For lower photon energies, the length is a critical factor, for example the limiting factor for brightness and flux at 1 keV is straight section length. Reducing the vacuum chamber gap would be beneficial across the board in terms of brightness and flux, although other effects will play a bigger role, *e.g.* impedance, beam-induced heating *etc*.

Note that choosing the operational energy for a synchrotron project is crucial to determining the construction and operating costs; higher energy leads to a higher cost, mainly driven by more powerful RF systems, as well as stronger magnets.

## Figures and Tables

**Figure 2:**
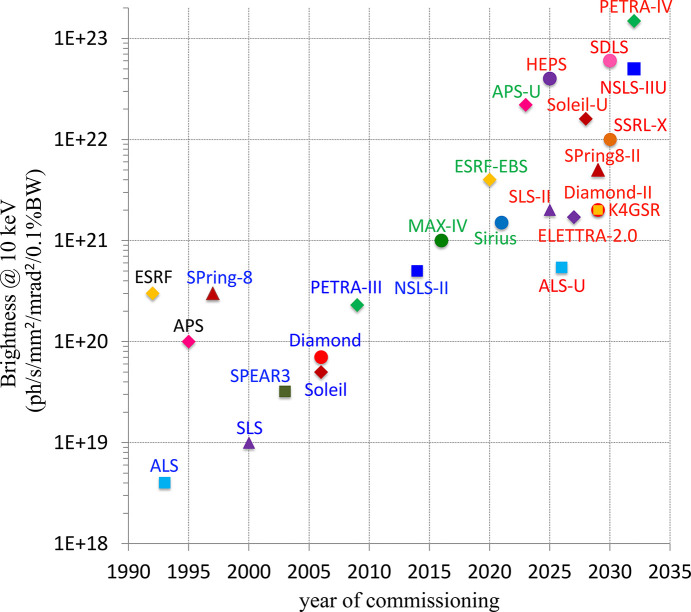
Evolution of brightness at 10 keV photon energy.

**Table 1:** Parameters of the light sources

Facility	Year	*E* (GeV)	*C* (m)	*I* (mA)	ɛ_*x*_ (pm)	ɛ_*y*_ (pm)	σ_δ_ (%)	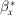 (m)†	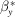 (m)†	References
ESRF	1992	6	844	200	3985	4	0.106	35.1	2.5	Raimondi *et al.* (2023 ▸), Ropert & Farvacque (2006 ▸)
ALS	1993	1.9	197	500	2000	30	0.097	21.2	1.7	Steier *et al.* (2018 ▸), Steier *et al.* (2014 ▸)
ELETTRA	1994	2	259	310	7000	70	0.080	7.0	2.6	Karantzoulis (2018 ▸), Wrulich (1988 ▸)
APS	1995	7	1104	100	2500	25	0.096	19.5	2.8	Hettel (2021 ▸), Sajaev *et al.* (2007 ▸)
SPring8	1997	8	1436	100	2400	4.8	0.109	31.2	5.0	Tanaka *et al.* (2024 ▸), Tanaka *et al.* (2016 ▸)
SLS	2000	2.4	288	400	5630	10	0.086	1.7	2.6	Boge (2002 ▸), Streun *et al.* (2018 ▸)
SPEAR3	2003	3	234	500	6700	10	0.097	9.0	5.3	Raimondi *et al.* (2024 ▸), Tian *et al.* (2022 ▸)
Soleil	2006	2.75	354	500	3700	37	0.116	4.7	2.0	Loulergue *et al.* (2018 ▸), Level *et al.* (2002 ▸)
Diamond	2006	3	562	300	2690	8	0.096	4.7	1.5	Ghasem *et al.* (2024 ▸), Suller (2002 ▸)
PETRA-III	2009	6	2304	120	1300	10	0.130	1.4	4.0	Schroer *et al.* (2022 ▸), Schroer *et al.* (2018 ▸)
NSLS-II	2014	3	792	500	950	8	0.082	1.8	1.2	Dierker (2007 ▸), Smaluk *et al.* (2019 ▸)
MAX-IV	2016	3	528	500	263	8	0.096	9.0	2.0	Leemann *et al.* (2009 ▸), Tavares *et al.* (2018 ▸)
ESRF-EBS	2020	6	844	200	133	1	0.094	6.9	2.7	Raimondi *et al.* (2023 ▸)
Sirius	2021	3	518	350	280	2.8	0.083	4.0	0.9	Liu *et al.* (2014 ▸)
APS-U	2024	6	1104	200	21	21	0.129	4.9	1.9	Hettel (2021 ▸), Borland *et al.* (2017 ▸)
HEPS	2025	6	1360	200	32	3.2	0.102	2.8	1.9	Jiao *et al.* (2018 ▸), Xu *et al.* (2023 ▸)
SLS-2	2025‡	2.4	290	400	98	10	0.103	3.5	2.0	Streun *et al.* (2018 ▸)
ALS-U	2026	2	197	500	55	55	0.080	2.2	2.6	Steier *et al.* (2018 ▸), Sun *et al.* (2017 ▸)
Diamond-II	2029‡	3.5	561	300	162	8	0.094	2.1	1.6	Ghasem *et al.* (2024 ▸)
Soleil-U	2028‡	2.75	353	500	50	50	0.086	1.1	1.1	Loulergue *et al.* (2018 ▸)
ELETTRA-2.0	2027	2.4	259	400	250	2.5	0.067	11.6	2.3	Karantzoulis (2018 ▸)
K4GSR	2028	4	799	400	58	5.8	0.120	8.6	2.5	Ko *et al.* (2022 ▸)
PETRA-IV	2032‡	6	2304	200	20	5	0.090	4.0	4.0	Schroer *et al.* (2022 ▸), Schroer *et al.* (2018 ▸)
SPring8-II	2030	6	1435	200	50	5	0.098	8.2	2.8	Tanaka *et al.* (2024 ▸), Tanaka *et al.* (2016 ▸)
SSRL-X	2030‡	4	587	400	58	12	0.073	2.0	2.0	Raimondi *et al.* (2024 ▸)
SDLS	2030‡	5	2190	300	8	8	0.088	2.0	2.0	Raimondi *et al.* (2024 ▸)
NSLS-IIU	2032‡	4	792	400	16	16	0.109	1.9	2.0	Song & Shaftan (2024 ▸)

† Beta functions in the centers of straight sections.

‡ The notional timeframe for the commissioning of some upgrade projects.
